# 1,10-Phenanthrolinium 2,3,4,5,6-penta­fluoro­benzoate–2,3,4,5,6-penta­fluoro­benzoic acid (1/2)

**DOI:** 10.1107/S160053680903801X

**Published:** 2009-09-26

**Authors:** Xiangdong Zhang, Yanmei Men, Xianghua Yan, Chunhua Ge, Yuxia Kong

**Affiliations:** a College of Chemistry, Liaoning University, Shenyang 110036, People’s Republic of China

## Abstract

In the title compound, C_12_H_9_N_2_^+^·C_7_F_5_O_2_^−^·2C_7_HF_5_O_2_, the cation and anion are linked by an N—H⋯O hydrogen bond. The neutral mol­ecules bond to the anion *via* O—H⋯O hydrogen bonds to form associations of one cation, one anion and two neutral mol­ecules. Inter­molecular C—H⋯O, C—H⋯F, F⋯F [shortest contact = 2.768 (8) Å], F⋯π [shortest contact = 3.148 (13) Å] and π–π [shortest centroid–centroid separation = 3.689 (5) Å] inter­actions further link the components to form a three-dimensional network.

## Related literature

For recent developments in the supra­molecular chemistry of fluorine-containing compounds, see: Chopra & Row (2008 ▶); Choudhury & Row (2004 ▶); Gdaniec *et al.* (2003 ▶); Kawahara *et al.* (2004 ▶); Mori & Matsumoo (2007 ▶); Reddy *et al.* (2004 ▶).
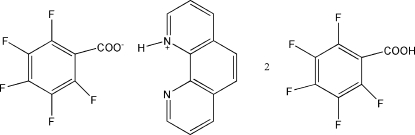


## Experimental

### 

#### Crystal data

C_12_H_9_N_2_^+^·C_7_F_5_O_2_^−^·2C_7_HF_5_O_2_
*M* *_r_* = 816.44
Triclinic, 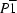

*a* = 9.288 (2) Å
*b* = 11.099 (3) Å
*c* = 15.723 (6) Å
α = 75.93 (3)°
β = 79.45 (2)°
γ = 87.14 (2)°
*V* = 1545.6 (8) Å^3^
*Z* = 2
Mo *K*α radiation
μ = 0.18 mm^−1^
*T* = 293 K
0.30 × 0.25 × 0.25 mm

#### Data collection

Bruker SMART CCD diffractometer
Absorption correction: multi-scan (*SADABS*; Bruker, 2001 ▶) *T*_min_ = 0.934, *T*_max_ = 0.952
6444 measured reflections
5387 independent reflections
1611 reflections with *I* > 2σ(*I*)
*R*_int_ = 0.075

#### Refinement

*R*[*F* ^2^ > 2σ(*F* ^2^)] = 0.078
*wR*(*F* ^2^) = 0.276
*S* = 0.86
5387 reflections
508 parameters
H-atom parameters constrained
Δρ_max_ = 0.33 e Å^−3^
Δρ_min_ = −0.39 e Å^−3^

### 

Data collection: *SMART* (Bruker, 2001 ▶); cell refinement: *SAINT* (Bruker, 2001 ▶); data reduction: *SAINT*; program(s) used to solve structure: *SHELXS97* (Sheldrick, 2008 ▶); program(s) used to refine structure: *SHELXL97* (Sheldrick, 2008 ▶); molecular graphics: *SHELXTL* (Sheldrick, 2008 ▶); software used to prepare material for publication: *SHELXL97*.

## Supplementary Material

Crystal structure: contains datablocks I, global. DOI: 10.1107/S160053680903801X/hb5105sup1.cif

Structure factors: contains datablocks I. DOI: 10.1107/S160053680903801X/hb5105Isup2.hkl

Additional supplementary materials:  crystallographic information; 3D view; checkCIF report

## Figures and Tables

**Table 1:** Hydrogen-bond geometry (Å, °)

*D*—H⋯*A*	*D*—H	H⋯*A*	*D*⋯*A*	*D*—H⋯*A*
N1—H1*A*⋯O1	0.86	2.17	2.876 (8)	139
O3—H3*A*⋯O1	0.82	1.72	2.526 (8)	166
O5—H5⋯O2	0.82	1.80	2.572 (10)	157
C7—H7⋯O6^i^	0.93	2.54	3.353 (12)	146
C8—H8⋯O4^ii^	0.93	2.57	3.391 (11)	147
C2—H2⋯F15^iii^	0.93	2.51	3.357 (10)	151
C10—H10⋯F7^iv^	0.93	2.54	3.375 (11)	149

Symmetry codes: (i) 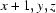; (ii) 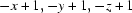; (iii) 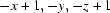; (iv) 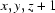.

**Fig. 1.**
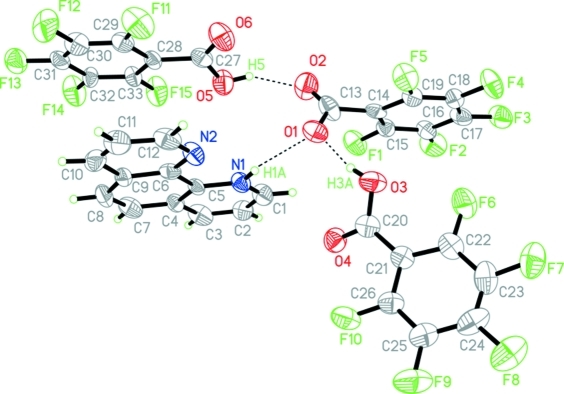
The structure of (I). Displacement ellipsoids are drawn at the 30% probability level.

## supplementary crystallographic information

### Comment

The research of supramolecular interaction based on the fluorine containing organic compound has been an area of explosive growth in recent years (Chopra & Row, 2008; Choudhury & Row, 2004; Gdaniec, *et al.*, 2003; Kawahara, Tsuzuki & Uchimaru, 2004; Mori & Matsumoo, 2007; Reddy *et al.*,  2004). Recent studies unravel the importance of weak but important of fluorine involving interactions in many crystal structures. The structure of the title complex is shown in Fig. 1. N—H···O and O—H···O bond one 1,10-phenanthrolinium 2,3,4,5,6-pentafluorobenzoate and two 2,3,4,5,6-pentafluorobenzoic acid molecules form a organic cluster. Additional nonclassical hydrogen bond of C—H···O, C—H···F (Table 1), F···F [F2···F14(1 - *x*, -*y*,1 - *z*), 2.768 (8) Å; F3···F13(*x*, *y*, -1 + *z*), 2.867 (8) Å; F5···F7(-*x*, 1 - *y*, -*z*), 2.935 (8) Å; F6···F13(*x*, *y*, -1 + *z*), 2.861 (8) Å] and F···π[F4···C24(-*x*, 1 - *y*, -*z*), 3.148 (13) Å; F9···C16 (1 - *x*, 1 - *y*, -*z*), 3.103 (11) Å; F12···C21(-*x*, 1 - *y*, 1 - *z*), 3.078 (10) Å; F13···C17(*x*, *y*, 1 + *z*), 3.101 (11) Å] are involved in the construction of the supramolecular three-dimensional network. π–π stacking also strengthen the stability of the structure {[*Cg*_1_···*Cg*_2_ (1 - *x*,1 - *y*,1 - *z*) 3.700 (5) Å; *Cg*_2_···*Cg*_3_ (1 - *x*,1 - *y*,1 - *z*) 3.689 (5) Å; *Cg*_2_···*Cg*_5_ 3.786 (5) Å; *Cg*_4_···*Cg*_4_ (1 - *x*,1 - *y*,-*z*) 3.763 (6) Å; *Cg*_5_···*Cg*_6_ (-*x*, -*y*, 1 - *z*) 3.891 (5) Å], *Cg*_1_ is the centroid of the N1/C1–C5 ring, *Cg*_2_ is the centroid of the N2/C6/C9—C12 ring, *Cg*_3_ is the centroid of the C4—C9 ring, *Cg*_4_ is the centroid of the C21—C16 ring, *Cg*_5_ is the centroid of the C28—C33 ring, *Cg*_6_ is the centroid of the C14—C19 ring, respectively}.

### Experimental

A solution of 1,10-phenanthroline (5 mmol) in ethanol (10 ml) was added into 2,3,4,5,6-pentafluorobenzoic acid (15 mmol) in ethanol (25 ml). The mixture was filtered. Colourless blocks of (I) were formed after one week by evaporation of the solvent at room temperature.

### Refinement

All H atoms were placed in calculated positions and included in a riding-model approximation, with C—H = 0.93 Å, O—H = 0.82 Å, N—H = 0.86 Å and *U*_iso_(H)= 1.2U_eq_(C), *U*_iso_(H)= 1.2U_eq_(N) or 1.5U_eq_(O).

### Crystal data

**Table:**

C_12_H_9_N_2_^+^·C_7_F_5_O_2_^−^·2C_7_HF_5_O_2_	*Z* = 2
*M**_r_* = 816.44	*F*(000) = 812
Triclinic, *P*1	*D*_x_ = 1.754 Mg m^−^^3^
Hall symbol: -P 1	Mo *K*α radiation, λ = 0.71073 Å
*a* = 9.288 (2) Å	Cell parameters from 76 reflections
*b* = 11.099 (3) Å	θ = 2.1–22.2°
*c* = 15.723 (6) Å	µ = 0.18 mm^−^^1^
α = 75.93 (3)°	*T* = 293 K
β = 79.45 (2)°	Block, colourless
γ = 87.14 (2)°	0.30 × 0.25 × 0.25 mm
*V* = 1545.6 (8) Å^3^	

### Data collection

**Table:**

Bruker SMART CCD diffractometer	5387 independent reflections
Radiation source: fine-focus sealed tube	1611 reflections with *I* > 2σ(*I*)
graphite	*R*_int_ = 0.075
ω scans	θ_max_ = 25.0°, θ_min_ = 2.1°
Absorption correction: multi-scan (*SADABS*; Bruker, 2001)	*h* = −11→1
*T*_min_ = 0.934, *T*_max_ = 0.952	*k* = −13→13
6444 measured reflections	*l* = −18→18

### Refinement

**Table:**

Refinement on *F*^2^	Primary atom site location: structure-invariant direct methods
Least-squares matrix: full	Secondary atom site location: difference Fourier map
*R*[*F*^2^ > 2σ(*F*^2^)] = 0.078	Hydrogen site location: inferred from neighbouring sites
*wR*(*F*^2^) = 0.276	H-atom parameters constrained
*S* = 0.86	*w* = 1/[σ^2^(*F*_o_^2^) + (0.1316*P*)^2^] where *P* = (*F*_o_^2^ + 2*F*_c_^2^)/3
5387 reflections	(Δ/σ)_max_ = 0.001
508 parameters	Δρ_max_ = 0.33 e Å^−^^3^
0 restraints	Δρ_min_ = −0.39 e Å^−^^3^

### Special details

**Table:**

Geometry. All e.s.d.'s (except the e.s.d. in the dihedral angle between two l.s. planes) are estimated using the full covariance matrix. The cell e.s.d.'s are taken into account individually in the estimation of e.s.d.'s in distances, angles and torsion angles; correlations between e.s.d.'s in cell parameters are only used when they are defined by crystal symmetry. An approximate (isotropic) treatment of cell e.s.d.'s is used for estimating e.s.d.'s involving l.s. planes.
Refinement. Refinement of *F*^2^ against ALL reflections. The weighted *R*-factor *wR* and goodness of fit *S* are based on *F*^2^, conventional *R*-factors *R* are based on *F*, with *F* set to zero for negative *F*^2^. The threshold expression of *F*^2^ > σ(*F*^2^) is used only for calculating *R*-factors(gt) *etc*. and is not relevant to the choice of reflections for refinement. *R*-factors based on *F*^2^ are statistically about twice as large as those based on *F*, and *R*- factors based on ALL data will be even larger.

### Fractional atomic coordinates and isotropic or equivalent isotropic displacement parameters (Å^2^)

**Table:**

	*x*	*y*	*z*	*U*_iso_*/*U*_eq_	
C1	0.4878 (9)	0.2131 (7)	0.3967 (6)	0.074 (2)	
H1	0.4624	0.1972	0.3460	0.088*	
C2	0.6122 (9)	0.1593 (7)	0.4257 (6)	0.070 (2)	
H2	0.6686	0.1063	0.3952	0.084*	
C3	0.6539 (9)	0.1832 (7)	0.4993 (7)	0.080 (3)	
H3	0.7385	0.1477	0.5187	0.096*	
C4	0.5663 (7)	0.2620 (6)	0.5443 (5)	0.0533 (19)	
C5	0.4400 (7)	0.3161 (6)	0.5110 (5)	0.0462 (17)	
C6	0.3436 (8)	0.3972 (6)	0.5566 (5)	0.054 (2)	
C7	0.5997 (9)	0.2977 (7)	0.6186 (7)	0.080 (3)	
H7	0.6849	0.2665	0.6394	0.096*	
C8	0.5155 (9)	0.3739 (8)	0.6606 (6)	0.074 (2)	
H8	0.5416	0.3937	0.7099	0.089*	
C9	0.3853 (8)	0.4250 (7)	0.6296 (5)	0.058 (2)	
C10	0.2887 (10)	0.5029 (7)	0.6736 (6)	0.076 (3)	
H10	0.3065	0.5211	0.7255	0.091*	
C11	0.1699 (11)	0.5500 (8)	0.6380 (7)	0.087 (3)	
H11	0.1080	0.6044	0.6642	0.105*	
C12	0.1388 (8)	0.5189 (7)	0.5633 (7)	0.077 (3)	
H12	0.0551	0.5521	0.5415	0.092*	
C13	0.1394 (9)	0.1546 (7)	0.3570 (7)	0.075 (3)	
C14	0.1375 (8)	0.1115 (6)	0.2753 (5)	0.056 (2)	
C15	0.2427 (8)	0.0300 (7)	0.2470 (6)	0.060 (2)	
C16	0.2420 (8)	−0.0126 (7)	0.1718 (6)	0.065 (2)	
C17	0.1343 (9)	0.0258 (7)	0.1219 (6)	0.070 (2)	
C18	0.0268 (8)	0.1056 (7)	0.1491 (5)	0.060 (2)	
C19	0.0311 (8)	0.1477 (7)	0.2225 (6)	0.059 (2)	
C20	0.3200 (10)	0.4593 (7)	0.1686 (7)	0.069 (2)	
C21	0.3384 (8)	0.5419 (7)	0.0810 (6)	0.064 (2)	
C22	0.2675 (9)	0.5264 (8)	0.0133 (7)	0.074 (2)	
C23	0.2855 (12)	0.6078 (11)	−0.0694 (8)	0.090 (3)	
C24	0.3716 (13)	0.7039 (10)	−0.0867 (8)	0.095 (4)	
C25	0.4436 (11)	0.7280 (10)	−0.0227 (8)	0.087 (3)	
C26	0.4280 (9)	0.6450 (8)	0.0577 (7)	0.074 (2)	
C27	0.0622 (10)	0.1652 (7)	0.6233 (6)	0.065 (2)	
C28	0.1027 (8)	0.1805 (6)	0.7065 (5)	0.0546 (19)	
C29	0.0191 (8)	0.2546 (7)	0.7561 (6)	0.064 (2)	
C30	0.0509 (9)	0.2739 (8)	0.8310 (7)	0.078 (3)	
C31	0.1650 (9)	0.2107 (7)	0.8679 (6)	0.070 (2)	
C32	0.2517 (8)	0.1351 (7)	0.8192 (5)	0.0565 (19)	
C33	0.2195 (7)	0.1205 (6)	0.7433 (5)	0.0541 (19)	
F1	0.3534 (5)	−0.0062 (4)	0.2915 (4)	0.0929 (16)	
F2	0.3421 (5)	−0.0938 (5)	0.1470 (4)	0.1060 (19)	
F3	0.1284 (5)	−0.0194 (4)	0.0509 (4)	0.0906 (16)	
F4	−0.0784 (5)	0.1431 (5)	0.0989 (4)	0.0996 (17)	
F5	−0.0761 (5)	0.2232 (4)	0.2477 (3)	0.0887 (16)	
F6	0.1787 (6)	0.4264 (5)	0.0310 (4)	0.1046 (18)	
F7	0.2095 (7)	0.5766 (6)	−0.1270 (4)	0.126 (2)	
F8	0.3868 (7)	0.7807 (6)	−0.1671 (5)	0.134 (2)	
F9	0.5289 (7)	0.8267 (6)	−0.0453 (5)	0.137 (3)	
F10	0.5005 (6)	0.6728 (4)	0.1161 (4)	0.1004 (18)	
F11	−0.0973 (5)	0.3169 (5)	0.7283 (4)	0.115 (2)	
F12	−0.0313 (6)	0.3457 (5)	0.8780 (4)	0.118 (2)	
F13	0.1964 (5)	0.2264 (5)	0.9440 (4)	0.0971 (17)	
F14	0.3613 (6)	0.0731 (5)	0.8556 (4)	0.112 (2)	
F15	0.3053 (5)	0.0431 (5)	0.7037 (4)	0.1061 (19)	
N1	0.4050 (6)	0.2857 (5)	0.4388 (4)	0.0610 (17)	
H1A	0.3259	0.3152	0.4203	0.073*	
N2	0.2233 (6)	0.4436 (5)	0.5217 (4)	0.0642 (18)	
O1	0.1633 (6)	0.2656 (4)	0.3520 (4)	0.0755 (16)	
O2	0.1327 (8)	0.0702 (6)	0.4259 (5)	0.103 (2)	
O3	0.1898 (6)	0.4156 (5)	0.2009 (4)	0.0847 (18)	
H3A	0.1870	0.3755	0.2524	0.127*	
O4	0.4205 (6)	0.4356 (5)	0.2115 (4)	0.0821 (18)	
O5	0.1662 (6)	0.1127 (6)	0.5748 (4)	0.0879 (18)	
H5	0.1327	0.0928	0.5354	0.132*	
O6	−0.0504 (7)	0.2012 (6)	0.5975 (5)	0.101 (2)	

### Atomic displacement parameters (Å^2^)

**Table:**

	*U*^11^	*U*^22^	*U*^33^	*U*^12^	*U*^13^	*U*^23^
C1	0.066 (6)	0.074 (5)	0.091 (7)	0.006 (5)	−0.007 (5)	−0.046 (5)
C2	0.067 (6)	0.062 (5)	0.083 (7)	0.005 (4)	−0.001 (5)	−0.032 (5)
C3	0.056 (5)	0.062 (5)	0.128 (9)	0.013 (4)	−0.018 (5)	−0.035 (6)
C4	0.049 (4)	0.055 (4)	0.059 (5)	0.002 (4)	−0.021 (4)	−0.012 (4)
C5	0.046 (4)	0.039 (4)	0.058 (5)	−0.007 (3)	−0.007 (4)	−0.021 (4)
C6	0.049 (4)	0.037 (4)	0.071 (6)	−0.008 (3)	0.002 (4)	−0.011 (4)
C7	0.070 (6)	0.059 (5)	0.116 (8)	−0.002 (4)	−0.022 (6)	−0.024 (5)
C8	0.081 (6)	0.079 (6)	0.076 (7)	−0.010 (5)	−0.033 (5)	−0.028 (5)
C9	0.057 (5)	0.064 (5)	0.059 (5)	−0.010 (4)	−0.004 (4)	−0.029 (4)
C10	0.086 (7)	0.071 (6)	0.072 (6)	−0.011 (5)	0.010 (5)	−0.032 (5)
C11	0.080 (7)	0.069 (6)	0.115 (9)	0.000 (5)	0.005 (6)	−0.044 (6)
C12	0.045 (5)	0.069 (5)	0.119 (8)	0.009 (4)	−0.003 (5)	−0.038 (6)
C13	0.092 (7)	0.039 (5)	0.108 (8)	0.004 (4)	−0.048 (6)	−0.023 (5)
C14	0.048 (4)	0.042 (4)	0.074 (6)	−0.017 (4)	−0.008 (4)	−0.004 (4)
C15	0.058 (5)	0.054 (5)	0.065 (6)	−0.005 (4)	−0.017 (4)	−0.006 (4)
C16	0.050 (5)	0.069 (5)	0.084 (7)	0.003 (4)	−0.009 (5)	−0.040 (5)
C17	0.065 (5)	0.059 (5)	0.093 (7)	−0.017 (4)	−0.003 (5)	−0.037 (5)
C18	0.061 (5)	0.060 (5)	0.062 (6)	−0.001 (4)	−0.016 (4)	−0.016 (4)
C19	0.056 (5)	0.055 (5)	0.070 (6)	0.003 (4)	−0.005 (4)	−0.026 (4)
C20	0.065 (6)	0.054 (5)	0.089 (8)	0.012 (5)	−0.007 (6)	−0.024 (5)
C21	0.056 (5)	0.056 (5)	0.082 (7)	0.016 (4)	−0.017 (5)	−0.016 (5)
C22	0.071 (6)	0.054 (5)	0.103 (8)	0.026 (5)	−0.026 (6)	−0.029 (6)
C23	0.099 (8)	0.085 (7)	0.098 (9)	0.049 (6)	−0.052 (7)	−0.028 (7)
C24	0.105 (9)	0.057 (6)	0.094 (9)	0.031 (6)	0.010 (7)	0.009 (6)
C25	0.072 (7)	0.079 (7)	0.095 (9)	0.010 (6)	−0.006 (6)	−0.003 (7)
C26	0.058 (5)	0.069 (6)	0.088 (8)	0.005 (5)	−0.009 (5)	−0.012 (6)
C27	0.064 (6)	0.050 (5)	0.082 (7)	−0.009 (4)	−0.017 (5)	−0.011 (4)
C28	0.060 (5)	0.047 (4)	0.063 (5)	−0.010 (4)	−0.015 (4)	−0.020 (4)
C29	0.050 (5)	0.061 (5)	0.082 (7)	0.006 (4)	−0.016 (5)	−0.017 (5)
C30	0.062 (6)	0.066 (5)	0.113 (9)	0.016 (4)	−0.008 (6)	−0.040 (6)
C31	0.063 (5)	0.065 (5)	0.092 (7)	−0.017 (4)	−0.005 (5)	−0.040 (5)
C32	0.052 (4)	0.076 (5)	0.048 (5)	0.011 (4)	−0.019 (4)	−0.022 (4)
C33	0.048 (4)	0.057 (4)	0.067 (6)	0.015 (4)	−0.023 (4)	−0.027 (4)
F1	0.076 (3)	0.093 (3)	0.123 (5)	0.017 (3)	−0.041 (3)	−0.038 (3)
F2	0.079 (3)	0.103 (4)	0.145 (5)	0.027 (3)	−0.007 (3)	−0.063 (4)
F3	0.088 (3)	0.091 (3)	0.107 (4)	−0.009 (3)	−0.008 (3)	−0.057 (3)
F4	0.087 (3)	0.110 (4)	0.124 (5)	0.021 (3)	−0.061 (3)	−0.041 (3)
F5	0.077 (3)	0.089 (3)	0.114 (4)	0.032 (3)	−0.026 (3)	−0.051 (3)
F6	0.124 (4)	0.093 (4)	0.115 (5)	0.002 (3)	−0.044 (4)	−0.043 (3)
F7	0.136 (5)	0.149 (5)	0.096 (5)	0.039 (4)	−0.041 (4)	−0.030 (4)
F8	0.129 (5)	0.119 (5)	0.120 (5)	0.052 (4)	−0.002 (4)	0.009 (4)
F9	0.113 (5)	0.102 (4)	0.166 (7)	−0.026 (4)	−0.003 (4)	0.015 (4)
F10	0.086 (4)	0.089 (4)	0.125 (5)	−0.023 (3)	−0.020 (3)	−0.015 (3)
F11	0.081 (4)	0.099 (4)	0.181 (6)	0.046 (3)	−0.055 (4)	−0.050 (4)
F12	0.109 (4)	0.134 (5)	0.132 (5)	0.053 (4)	−0.014 (4)	−0.086 (4)
F13	0.097 (4)	0.096 (4)	0.113 (5)	−0.003 (3)	−0.016 (3)	−0.053 (3)
F14	0.091 (4)	0.132 (4)	0.143 (5)	0.055 (3)	−0.066 (4)	−0.069 (4)
F15	0.088 (3)	0.132 (4)	0.129 (5)	0.057 (3)	−0.039 (3)	−0.087 (4)
N1	0.053 (4)	0.064 (4)	0.074 (5)	0.003 (3)	−0.013 (3)	−0.030 (4)
N2	0.054 (4)	0.061 (4)	0.091 (5)	0.012 (3)	−0.023 (4)	−0.038 (4)
O1	0.076 (4)	0.048 (3)	0.103 (5)	−0.004 (3)	−0.018 (3)	−0.018 (3)
O2	0.148 (6)	0.071 (4)	0.093 (6)	−0.012 (4)	−0.025 (5)	−0.020 (4)
O3	0.070 (4)	0.074 (4)	0.101 (6)	−0.013 (3)	−0.014 (4)	−0.002 (4)
O4	0.073 (4)	0.097 (4)	0.077 (4)	0.010 (3)	−0.023 (3)	−0.017 (3)
O5	0.085 (4)	0.105 (5)	0.086 (5)	0.012 (4)	−0.025 (4)	−0.041 (4)
O6	0.082 (4)	0.111 (5)	0.126 (6)	0.020 (4)	−0.056 (4)	−0.033 (4)

### Geometric parameters (Å, °)

**Table:**

C1—N1	1.299 (9)	C18—C19	1.354 (10)
C1—C2	1.376 (11)	C18—F4	1.354 (8)
C1—H1	0.9300	C19—F5	1.336 (8)
C2—C3	1.371 (12)	C20—O4	1.231 (10)
C2—H2	0.9300	C20—O3	1.292 (9)
C3—C4	1.394 (10)	C20—C21	1.442 (11)
C3—H3	0.9300	C21—C26	1.384 (11)
C4—C7	1.411 (11)	C21—C22	1.399 (11)
C4—C5	1.419 (9)	C22—F6	1.359 (9)
C5—N1	1.357 (9)	C22—C23	1.377 (13)
C5—C6	1.457 (9)	C23—C24	1.310 (13)
C6—N2	1.358 (9)	C23—F7	1.359 (11)
C6—C9	1.382 (10)	C24—F8	1.329 (11)
C7—C8	1.334 (11)	C24—C25	1.389 (14)
C7—H7	0.9300	C25—F9	1.322 (11)
C8—C9	1.427 (10)	C25—C26	1.359 (12)
C8—H8	0.9300	C26—F10	1.333 (10)
C9—C10	1.421 (10)	C27—O6	1.206 (9)
C10—C11	1.353 (12)	C27—O5	1.320 (9)
C10—H10	0.9300	C27—C28	1.476 (11)
C11—C12	1.382 (13)	C28—C29	1.385 (10)
C11—H11	0.9300	C28—C33	1.387 (9)
C12—N2	1.324 (9)	C29—C30	1.332 (11)
C12—H12	0.9300	C29—F11	1.338 (8)
C13—O2	1.242 (10)	C30—F12	1.336 (9)
C13—O1	1.244 (8)	C30—C31	1.381 (11)
C13—C14	1.479 (12)	C31—F13	1.333 (10)
C14—C15	1.386 (10)	C31—C32	1.400 (10)
C14—C19	1.388 (10)	C32—C33	1.328 (10)
C15—F1	1.340 (8)	C32—F14	1.338 (8)
C15—C16	1.376 (11)	C33—F15	1.331 (7)
C16—F2	1.336 (8)	N1—H1A	0.8600
C16—C17	1.372 (11)	O3—H3A	0.8200
C17—F3	1.340 (9)	O5—H5	0.8200
C17—C18	1.379 (10)		
			
N1—C1—C2	121.3 (8)	F4—C18—C17	118.3 (8)
N1—C1—H1	119.3	F5—C19—C18	118.4 (7)
C2—C1—H1	119.3	F5—C19—C14	118.3 (7)
C3—C2—C1	120.6 (8)	C18—C19—C14	123.2 (7)
C3—C2—H2	119.7	O4—C20—O3	121.9 (9)
C1—C2—H2	119.7	O4—C20—C21	122.1 (9)
C2—C3—C4	118.4 (8)	O3—C20—C21	115.9 (8)
C2—C3—H3	120.8	C26—C21—C22	114.3 (9)
C4—C3—H3	120.8	C26—C21—C20	122.0 (9)
C3—C4—C7	124.0 (8)	C22—C21—C20	123.6 (9)
C3—C4—C5	118.8 (7)	F6—C22—C23	119.9 (9)
C7—C4—C5	117.1 (7)	F6—C22—C21	117.7 (9)
N1—C5—C4	119.0 (6)	C23—C22—C21	122.4 (10)
N1—C5—C6	120.0 (6)	C24—C23—F7	126.0 (12)
C4—C5—C6	120.9 (7)	C24—C23—C22	119.9 (10)
N2—C6—C9	125.2 (7)	F7—C23—C22	114.1 (11)
N2—C6—C5	117.5 (7)	C23—C24—F8	118.5 (13)
C9—C6—C5	117.3 (7)	C23—C24—C25	121.5 (11)
C8—C7—C4	123.4 (8)	F8—C24—C25	119.9 (12)
C8—C7—H7	118.3	F9—C25—C26	124.2 (11)
C4—C7—H7	118.3	F9—C25—C24	117.9 (11)
C7—C8—C9	119.7 (8)	C26—C25—C24	117.8 (11)
C7—C8—H8	120.1	F10—C26—C25	115.0 (9)
C9—C8—H8	120.1	F10—C26—C21	121.0 (9)
C6—C9—C10	116.3 (8)	C25—C26—C21	123.9 (10)
C6—C9—C8	121.4 (7)	O6—C27—O5	121.3 (9)
C10—C9—C8	122.2 (8)	O6—C27—C28	125.2 (9)
C11—C10—C9	118.1 (9)	O5—C27—C28	113.4 (7)
C11—C10—H10	121.0	C29—C28—C33	114.8 (7)
C9—C10—H10	121.0	C29—C28—C27	120.6 (7)
C10—C11—C12	121.4 (8)	C33—C28—C27	124.6 (7)
C10—C11—H11	119.3	C30—C29—F11	115.6 (8)
C12—C11—H11	119.3	C30—C29—C28	123.6 (7)
N2—C12—C11	122.6 (8)	F11—C29—C28	120.8 (8)
N2—C12—H12	118.7	C29—C30—F12	122.7 (9)
C11—C12—H12	118.7	C29—C30—C31	120.6 (8)
O2—C13—O1	124.7 (9)	F12—C30—C31	116.4 (9)
O2—C13—C14	114.5 (7)	F13—C31—C30	121.6 (8)
O1—C13—C14	120.3 (9)	F13—C31—C32	121.5 (8)
C15—C14—C19	115.4 (7)	C30—C31—C32	116.8 (8)
C15—C14—C13	121.5 (7)	C33—C32—F14	122.3 (7)
C19—C14—C13	123.1 (7)	C33—C32—C31	120.9 (7)
F1—C15—C16	117.7 (7)	F14—C32—C31	116.7 (7)
F1—C15—C14	119.7 (7)	C32—C33—F15	116.3 (6)
C16—C15—C14	122.5 (8)	C32—C33—C28	123.0 (7)
F2—C16—C17	118.5 (7)	F15—C33—C28	120.7 (7)
F2—C16—C15	121.5 (8)	C1—N1—C5	121.8 (7)
C17—C16—C15	119.9 (7)	C1—N1—H1A	119.1
F3—C17—C16	120.5 (7)	C5—N1—H1A	119.1
F3—C17—C18	120.4 (8)	C12—N2—C6	116.4 (7)
C16—C17—C18	118.9 (8)	C20—O3—H3A	109.5
C19—C18—F4	121.7 (7)	C27—O5—H5	109.5
C19—C18—C17	120.0 (8)		
			
N1—C1—C2—C3	−1.3 (13)	C20—C21—C22—F6	−1.3 (11)
C1—C2—C3—C4	0.7 (13)	C26—C21—C22—C23	−0.3 (11)
C2—C3—C4—C7	−177.5 (8)	C20—C21—C22—C23	179.1 (7)
C2—C3—C4—C5	−1.4 (12)	F6—C22—C23—C24	−179.7 (8)
C3—C4—C5—N1	2.6 (10)	C21—C22—C23—C24	0.0 (13)
C7—C4—C5—N1	179.1 (7)	F6—C22—C23—F7	−0.6 (11)
C3—C4—C5—C6	178.8 (7)	C21—C22—C23—F7	179.1 (7)
C7—C4—C5—C6	−4.7 (10)	F7—C23—C24—F8	1.0 (14)
N1—C5—C6—N2	−2.6 (9)	C22—C23—C24—F8	180.0 (7)
C4—C5—C6—N2	−178.8 (6)	F7—C23—C24—C25	179.5 (8)
N1—C5—C6—C9	−179.9 (7)	C22—C23—C24—C25	−1.5 (14)
C4—C5—C6—C9	4.0 (10)	C23—C24—C25—F9	179.9 (8)
C3—C4—C7—C8	179.5 (8)	F8—C24—C25—F9	−1.7 (13)
C5—C4—C7—C8	3.3 (12)	C23—C24—C25—C26	3.3 (14)
C4—C7—C8—C9	−0.9 (13)	F8—C24—C25—C26	−178.2 (7)
N2—C6—C9—C10	3.7 (11)	F9—C25—C26—F10	3.3 (13)
C5—C6—C9—C10	−179.2 (6)	C24—C25—C26—F10	179.6 (8)
N2—C6—C9—C8	−178.6 (7)	F9—C25—C26—C21	179.9 (8)
C5—C6—C9—C8	−1.5 (11)	C24—C25—C26—C21	−3.8 (13)
C7—C8—C9—C6	0.1 (12)	C22—C21—C26—F10	178.7 (7)
C7—C8—C9—C10	177.6 (8)	C20—C21—C26—F10	−0.7 (11)
C6—C9—C10—C11	−4.2 (11)	C22—C21—C26—C25	2.3 (12)
C8—C9—C10—C11	178.1 (8)	C20—C21—C26—C25	−177.1 (8)
C9—C10—C11—C12	3.1 (13)	O6—C27—C28—C29	−9.0 (12)
C10—C11—C12—N2	−1.1 (15)	O5—C27—C28—C29	167.5 (7)
O2—C13—C14—C15	−58.5 (11)	O6—C27—C28—C33	168.2 (8)
O1—C13—C14—C15	113.5 (8)	O5—C27—C28—C33	−15.3 (11)
O2—C13—C14—C19	120.7 (9)	C33—C28—C29—C30	3.6 (12)
O1—C13—C14—C19	−67.2 (11)	C27—C28—C29—C30	−178.9 (8)
C19—C14—C15—F1	177.2 (7)	C33—C28—C29—F11	−179.6 (7)
C13—C14—C15—F1	−3.5 (11)	C27—C28—C29—F11	−2.1 (11)
C19—C14—C15—C16	−0.1 (11)	F11—C29—C30—F12	3.1 (13)
C13—C14—C15—C16	179.1 (8)	C28—C29—C30—F12	−179.9 (7)
F1—C15—C16—F2	4.5 (12)	F11—C29—C30—C31	177.3 (7)
C14—C15—C16—F2	−178.1 (7)	C28—C29—C30—C31	−5.7 (14)
F1—C15—C16—C17	−177.3 (7)	C29—C30—C31—F13	−178.3 (8)
C14—C15—C16—C17	0.1 (12)	F12—C30—C31—F13	−3.8 (12)
F2—C16—C17—F3	1.4 (12)	C29—C30—C31—C32	5.4 (13)
C15—C16—C17—F3	−176.9 (7)	F12—C30—C31—C32	179.9 (7)
F2—C16—C17—C18	177.3 (7)	F13—C31—C32—C33	−179.8 (8)
C15—C16—C17—C18	−1.0 (12)	C30—C31—C32—C33	−3.5 (12)
F3—C17—C18—C19	177.9 (7)	F13—C31—C32—F14	4.3 (11)
C16—C17—C18—C19	2.0 (12)	C30—C31—C32—F14	−179.4 (7)
F3—C17—C18—F4	−4.4 (11)	F14—C32—C33—F15	−1.9 (12)
C16—C17—C18—F4	179.7 (7)	C31—C32—C33—F15	−177.5 (7)
F4—C18—C19—F5	3.4 (11)	F14—C32—C33—C28	177.4 (7)
C17—C18—C19—F5	−178.9 (7)	C31—C32—C33—C28	1.8 (12)
F4—C18—C19—C14	−179.8 (7)	C29—C28—C33—C32	−1.6 (11)
C17—C18—C19—C14	−2.2 (12)	C27—C28—C33—C32	−179.0 (8)
C15—C14—C19—F5	178.0 (6)	C29—C28—C33—F15	177.6 (7)
C13—C14—C19—F5	−1.3 (11)	C27—C28—C33—F15	0.3 (11)
C15—C14—C19—C18	1.2 (11)	C2—C1—N1—C5	2.7 (12)
C13—C14—C19—C18	−178.1 (8)	C4—C5—N1—C1	−3.4 (10)
O4—C20—C21—C26	−34.9 (11)	C6—C5—N1—C1	−179.6 (7)
O3—C20—C21—C26	142.3 (8)	C11—C12—N2—C6	0.4 (12)
O4—C20—C21—C22	145.8 (8)	C9—C6—N2—C12	−1.8 (11)
O3—C20—C21—C22	−37.0 (11)	C5—C6—N2—C12	−178.8 (6)
C26—C21—C22—F6	179.3 (6)		

### Hydrogen-bond geometry (Å, °)

**Table:**

*D*—H···*A*	*D*—H	H···*A*	*D*···*A*	*D*—H···*A*
N1—H1A···O1	0.86	2.17	2.876 (8)	139
O3—H3A···O1	0.82	1.72	2.526 (8)	166
O5—H5···O2	0.82	1.80	2.572 (10)	157
C7—H7···O6^i^	0.93	2.54	3.353 (12)	146
C8—H8···O4^ii^	0.93	2.57	3.391 (11)	147
C2—H2···F15^iii^	0.93	2.51	3.357 (10)	151
C10—H10···F7^iv^	0.93	2.54	3.375 (11)	149

Symmetry codes:  (i) *x*+1, *y*, *z*; (ii) −*x*+1, −*y*+1, −*z*+1; (iii) −*x*+1, −*y*, −*z*+1; (iv) *x*, *y*, *z*+1.
